# How to Optimize Treatment With Ustekinumab in Inflammatory Bowel Disease: Lessons Learned From Clinical Trials and Real-World Data

**DOI:** 10.3389/fmed.2021.640813

**Published:** 2021-01-28

**Authors:** Ana Gutiérrez, Iago Rodríguez-Lago

**Affiliations:** ^1^Gastroenterology Department, Centro de Investigación Biomédica en Red de Enfermedades Hepáticas y Digestivas, Hospital General Universitario de Alicante, Alicante, Spain; ^2^Gastroenterology Department, Hospital de Galdakao, Biocruces Bizkaia Health Research Institute, Galdakao, Spain

**Keywords:** Crohn's disease, predictive factors, ulcerative colitis, ustekinumab, biological therapy

## Abstract

Ustekinumab is a fully human IgG1 monoclonal antibody that has been approved for the treatment of moderate to severe Crohn's disease, and more recently moderate to severe ulcerative colitis. It binds with high affinity to the p40 subunit of human interleukin-12 and 23. This mechanism of action prevents the bioactivity of both interleukins, thus precluding their interaction with the cell surface receptor protein. The pivotal clinical trials (UNITI-1, UNITI-2 and IM-UNITI) demonstrated its clinical efficacy and safety, in naïve patients and also in those previously exposed to immunosuppressants and/or biologics. There is now an extensive experience with its use worldwide, corroborating its favorable profile even in patients with refractory disease. However, the number of medical treatment options available in inflammatory bowel disease are still limited. Hence, we should prioritize the treatments that have a greater probability of response in an individual patient. Our aim was to review and summarize all the available literature regarding the potential predictors of response to ustekinumab that can increase the success rate with this therapy in clinical practice.

## Introduction: Do we Need Predictive Factors in Crohn'S Disease?

Inflammatory bowel diseases (IBD)—a term including both ulcerative colitis (UC) and Crohn's disease (CD)— are two chronic, disabling conditions causing an uncontrolled inflammatory process in the gastrointestinal tract, with a relapsing and remitting course (1, 2). It is considered that IBD appears in genetically predisposed subjects after the interaction with diverse environmental factors, therefore it is described as a complex disease where there is an interaction between multiple factors that has not been fully elucidated so far. The interaction between luminal antigens and the mucosal immune system seems to be crucial and mediated through an increased intestinal permeability, at least during the early stages of the disease (3). This interaction may trigger an abnormal and uncontrolled inflammatory response in susceptible individuals, leading to progressive bowel damage and symptomatic disease (4). Due to our increased knowledge of the immunological disturbances observed in these patients, new treatment options have been developed in recent years (5). Over the past 20 years, tumor necrosis factor (TNF)-antagonists have transformed the medical management of IBD due to their ability to induce a complete control of symptoms, induce mucosal healing in a significant proportion of patients, and reduce the long-term requirements of surgery and hospitalization (6–8). Despite their impact in the paradigm of disease control, many challenges remain: around two-thirds of IBD patients demonstrate short-term clinical response to anti-TNF therapy and ~40% of patients who initially improve subsequently lose response (9, 10). These data about the efficacy should be added to the potential adverse events associated to anti-TNF therapy, that underlines the urgent need of alternative therapeutic options targeting new disease pathways for refractory patients. In recent years, the experience with new biologics blocking leukocyte migration mediated through integrins—vedolizumab—or the immune pathways regulated by interleukin (IL)-12/23—ustekinumab (UST)—have increased the chance to obtain better disease control and improve quality of life. Hence, these new therapeutic options imply a greater probability of inducing disease remission in difficult-to-treat patients. Despite this important progress, the selection of first-line biologic therapy seems to be crucial, as it has consistently been shown that there is a stepwise reduced response rate with each subsequent biologic therapy (11, 12). Contrary to the aforementioned steps toward disease control, many regulatory authorities have approved UST only after anti-TNF failure, which significantly reduces the overall efficacy of the drug.

Taking into consideration that many new drugs involving other mechanisms of action are still to come, personalized medicine will gain importance in the near future (13, 14). This is a new concept in our field, but many new findings associated to the ability to predict response, relapse and even adverse events by using clinical data and biomarkers will allow us to choose the best drug, for an individual patient at the right time (15, 16). Several factors have been linked to the response to TNF blockade in IBD, including clinical factors, pharmacokinetics, biochemical markers, pharmacogenomics, microbiome signatures, metabolic compounds and mucosal markers (17, 18). While there are significant advances allowing a better identification of patients more likely to respond to anti-TNFs, including also a more profound understanding of its pharmacokinetics, few studies have investigated predictive factors of therapeutic efficacy to UST that may improve the probability of response and long-term benefit. This review will discuss all the possible factors and biomarkers associated to the initial and long-term response to UST in CD.

## The Unmet Needs With USTEKINUMAB: Efficacy in Randomized Clinical Trials and Observational Cohorts

### Clinical Trials

UST is a fully human immunoglobulin G1 monoclonal antibody that blocks the p40 subunit of IL-12 and IL-23, precluding cytokine-mediated cellular activation. IL-23 promotes the differentiation of naïve T cells into Th17 phenotype, whereas IL-12 regulates the Th1 polarization. The downstream effect of the IL-12/23 blockade is the neutralization of human IL-12 and IL-23-mediated cell signaling, cell activation, and cytokine production involved in the pathogenesis of CD (19). UST has demonstrated its efficacy inducing response and remission in CD patients in randomized clinical trials and also in real-life studies. The UNITI study, a phase III multicenter, double-blind, placebo-controlled randomized clinical trial included an induction (UNITI-1 and 2) and a maintenance phase (IM-UNITI) (20). Patients started UST after primary non-response, loss of response or intolerance to anti-TNF agents (UNITI-1), but also failure or severe adverse events during conventional therapy with immunosuppressants or steroids (UNITI-2). The primary aim—defined as a reduction in the Crohn's Disease Activity Index [CDAI] ≤100 or CDAI <150 at week 6—was achieved by 22, 34, and 34% in the placebo, UST 130 mg and UST 6 mg/kg groups, respectively, in the UNITI-1 trial (714 patients) and 29, 52, and 56% in the UNITI-2 (628 patients). In the IM-UNITI study, including 397 responders during the induction, the primary endpoint—clinical remission (CDAI <150) at week 44—was achieved by 36, 49, and 53% in the placebo, UST 90 mg q12w and 90 mg q8w arms, respectively. Long-term data from the IM-UNITI study show that 62 and 70% of patients in the q12w and q8w arms were in clinical remission at week 152, respectively (21). A treat-to-target approach based on endoscopic findings at week 16 has been evaluated with UST in the STARDUST trial (NCT03107793). This is the first randomized trial evaluating the efficacy of UST under a dose adjustment strategy based on biomarkers (fecal calprotectin and C-reactive protein) and symptoms (CDAI), compared with a standard, clinically-driven approach. Preliminary results have been presented at United European Gastroenterology Week 2020, were the treat-to-target strategy showed a numerically higher endoscopic response, but there were no clear differences between both treatment arms.

### Real-World Data

Additionally, several open-label observational cohort studies have also assessed and confirmed the efficacy and safety of UST for CD in clinical practice (22–41). Although these real-world studies have some obvious advantages over randomized clinical trials (as they reflect real clinical practice and in clinical scenarios where patients will not fulfill the rigorous inclusion criteria of clinical trials), their limitations need to be taken into account. Real-life studies are limited usually by smaller sample size, more limited follow-up, and are prone to bias due to their outcomes and frequent retrospective design. Nonetheless, real-world studies are an important source of information in addition to the results of clinical trials. Based on these assumptions, a recent multicenter retrospective Spanish study including 407 patients observed that 57 and 64% of patients with active disease starting UST achieved clinical remission at weeks 26 and 52, respectively (30, 31). Fecal calprotectin normalization was observed in 44 and 54% of patients at weeks 26 and 52, while C-reactive protein returned to normal in 36 and 37% of patients at the same time points, respectively. Biemans et al. recently reported results from the nationwide prospective observational Dutch cohort (40). This study included 221 CD patients, where corticosteroid-free clinical remission rates at weeks 24 and 52 were 38 and 37%, respectively. In conclusion, clinical trials and real-world studies have demonstrated that UST is safe and effective for the induction and maintenance of response and remission of refractory CD patients, but a significant proportion still fails to obtain the strict endpoints that should be regarded as our goals as steroid-free clinical remission and mucosal healing. Therefore, we will discuss the clinical factors and biomarkers that have been associated with a higher probability of clinical benefit with UST. As the IBD drug pipeline is still limited, the identification of predictive factors should be carefully considered as it may help us to enhance the probability of achieving disease remission.

## Predictive Factors Associated to Ustekinumab Response

### Clinical Factors

There are many aspects that influence the response to UST, but there have been no definite characteristics associated to a certain patient profile that may show a better response so far. However, some patient or disease-related aspects may help us to decide that anti-interleukin therapy is the best option in an individual patient (Table 1). Age is one of the most important factors, and it may be expected to plays an important role in the prognosis and treatment outcomes of CD. Nevertheless, some age subgroups of patients are usually underrepresented in most of the clinical trials and observational cohorts. Previous descriptions of the management of elderly IBD patients show that they usually receive steroids, immunosuppressants or anti-TNF agents less frequently (45). Though, regarding anti-TNF therapy there is no clear evidence about the influence of age on the response to these drugs (18). Data with UST is still limited, but in the 6 mg/kg and 130 mg treatment arm in the UNITI-1 trial, younger patients showed increased rates of clinical response at week 6 compared to placebo [odds ratio (OR), 2.4; confidence interval (CI) 95%, 1.3–4.3 and OR, 2.7; CI 95%, 1.5–4.9, respectively] (20). Only one additional retrospective case series has demonstrated that age influences treatment outcomes with UST (37). Here, Casas Deza et al. observed that older age was associated with reduced clinical response rates after 16 weeks of therapy. However, there are no consistent data across the remaining studies suggesting a different efficacy across different age groups (18). Hence, older patients may show a reduced clinical response to UST at least in the short-term, but data about treatment persistence and more robust outcomes are needed to confirm this relationship.

**Table 1:** Predictive factors of response in observational studies in patients with Crohn's disease.

						**Predictive factors**	
**References**	**Study design**	**UST dosing**	**Clinical scenario**	**No patients**	**Endpoint**	**Positive association**	**Inverse association**
Kopylov et al. (22)	Retrospective	Sc	CD refractory to at least one anti-TNF	38	Clinical response	-	-
Wils et al. (24)	Retrospective	Sc	CD refractory to immunosuppressants and anti-TNF	122	Clinical benefit at 3 months	Concomitant IM (OR 5.43; 95% CI 1.14–25.77)	-
Khorrami et al. (23)	Retrospective	Sc	CD refractory or intolerant to at least one anti-TNF	116	Clinical benefit	Previous intestinal resection (OR 2.09; 95% CI 1.16–3.79)	Initial response (OR 0.16; 95% CI 0.09–0.31) Previous use ≥2 IM (OR 0.5; 95% CI 0.28-0.88)
Harris et al. (28)	Retrospective	Sc	Complicated CD refractory to anti-TNF	45	Clinical response	-	-
Ma et al. (25)	Retrospective	Sc (89%)Iv (11%)	CD failing anti-TNF therapy	167	Steroid-free clinical response and remission	**Clinical response at 6 months** Ileocolonic disease (OR 2.41; 95% CI 1.01–5.79)	**Clinical response at 6 months** Harvey-Bradshaw index ≥ 7 (OR 0.26; 95% 0.11–0.61) Stricturing disease (OR 0.29; 95% 0.12–0.72) Immunomodulators at induction (OR 0.37; 95% CI 0.15–0.89)
Ma et al. (26)	Retrospective	Sc (88%)Iv (12%)	Primary clinical steroid-free response to UST	104	Loss of response among primary responders to UST	Harvey-Bradshaw index ≥ 7 (OR 4.63; 95% 1.64–13.11)Stricturing phenotype (OR 2.77; 95% 1.1–7.01)	Concomitant IM (OR 0.41; 95% 0.17–0.97) Colonic disease (OR 0.33; 95% 0.11–0.98) Ileocolonic (OR 0.26;95% 0.1–0.68)
Greenup et al. (27)	Retrospective	Sc	Real-world experience	73	Symptomatic response at 3, 3–12 and >12 months	Type of anti-TNF non-response: Primary non-response vs. secondary loss of response or intolerance (OR 17.33; 95% CI 2.34–128.47, and OR 26.56; 95% CI 3.46–203.62, respectively)	-
Wils et al. (38)	Retrospective	Sc	Real-world experience	88	Failure-free persistence in initial responders	**-**	**-**
Iborra et al. (30)	Retrospective	Iv induction	Luminal CD refractory to conventional therapy	305	Clinical remission at week 14	-	No of previous anti-TNF (OR 0.67; 95% CI 0.44–0.95) Endoscopic severity (OR 0.08; 95% CI 0.01–0.37) Intolerance to last anti-TNF vs. primary or secondary failure (OR 0.66; 95% CI 1.13–6.30)
Iborra et al. (31)	Retrospective	Iv induction	Moderate-severe CD and no response or insufficient response to conventional therapy	407	Remission and clinical remission at week 26 and 52	**Clinical remission at week 26** Response at week 14 (OR 9.90 95% CI 4.91–20.86)**Clinical remission at week 52**Response at week 14 (OR 8.45; 95% CI 3.97–18.8)	**Clinical remission at week 26** No of previous anti-TNF (OR 0.53; 95% CI 0.37–0.75) Colonic (OR 0.34; 95% CI 0.16–0.69) Ileocolonic (OR 0.56; 95% CI 0.32–0.96) **Clinical remission at week 52** No of previous anti-TNF (OR 0.52; 95% CI 0.35–0.78) Severe endoscopic activity (OR 0.35; 95% CI 0.16–0.71)
Harris et al. (29)	Retrospective	Iv induction	Clinically active CD	84	Clinical response and drug persistence	-	-
Murate et al. (33)	Prospective	Iv induction	Moderate-severe CD	22	Clinical response at 24 weeks	Higher TNF-α concentration (cut-off 19.58 pg/ml; AUROC 0.819)	Lower SES-CD at baseline (cut-off <13; AUROC = 0.757)
Liefferinckx et al. (32)	Prospective	Iv induction	CD refractory to anti-TNF therapy	152	Clinical response	**Clinical response** Colonic (OR 3.5; 95% CI 1.34–9.41)	**Clinical remission** Body mass index <18 (OR 0.28; 95% CI 0.09–0.87)
Bar-Gil et al. (34)	Prospective	Iv induction	Active CD	106	Clinical response at week 24	-	-
Bennet et al. (42)	Prospective	Sc (95%)Iv (5%)	Moderate-severe CD refractory to anti-TNF and/or vedolizumab	96	C-reactive protein, clinical activity and endoscopy	-	-
Saldaña Duenas et al. (36)	Prospective	Iv induction	Real-world experience in refractory CD	61	Clinical response and remission at week 16, 24, and 52	-	-
Casas Deza et al. (37)	Retrospective	Iv (83%)Sc (17%)	Real-world experience in refractory CD	69	Clinical disease activity at week 16	**Clinical response at week 16** Reason to stop prior biologic, adverse events (OR 96; CI 97.5% 10.15–1,273) or secondary loss of response (OR 7.07; 97.5% CI 1.22–48.02)	**Clinical response at week 16** Age (OR 0.95; 97.5% CI 0.90–0.99) Smoking habits (OR 0.19; 97.5% CI 0.04–0.78)
Hoffmann et al. (35)	Retrospective	Iv	Real-world experience in refractory CD	68	Steroid-free clinical remission or response at week 24	**-**	**Steroid free-clinical response** Male (OR 0.11; 95% CI 0.02–0.61) Steroids at baseline (OR 0.071; 95% CI 0.011–0.464) Extraintestinal manifestations (OR 0.119; 95% CI 0.022–0.636)
Kubesch et al. (39)	Retrospective	Iv induction	Real-world experience	106	Clinical and biochemical remission at week 48	Remission at week 8 (OR 4.75; 95% CI 1.21–18.58)Response at week 16 (OR 10.52; 95% CI 2.27–48.75)	Male gender (OR 0.26; 95% CI 0.08–0.88) Penetrating behavior (OR 0.25; 95% CI 0.07–0.89)
Biemans et al. (40)	Prospective	Iv induction	Real-world experience	221	Corticosteroid-free clinical remission at week 52	-	Body mass index (OR 0.91; 95% CI 0.83–1.00)
Li et al. (43)	Clinical trial (UNITI-1, UNITI-2 and IM-UNITI)	Iv induction	Moderate-severe CD	251	Overall Global Histology Activity Score at week 8	-	Baseline total SES-CD (OR 0.18; 95% 0.042–0.321) Baseline Overall Global Histology Activity Score (OR 0.374; 95% CI 0.213–0.535)
Waljee et al. (44)	Clinical trial (UNITI-1, UNITI-2 and IM-UNITI)	Iv induction	Moderate-severe active CD enrolled in pivotal RCT	401	Clinical and biochemical remission beyond week 42	**Predictors during induction (week 8)** CRP at baseline, week 3, 6, and 8	Baseline CRP cut-off 14.65 mg/L (AUROC 0.67; 95% CI 0.61–0.74)
						Serum UST to CRP ratio at week 3 and 6Albumin at week 8**Pragmatic modeling** Week-6 albumin to CRP ratio >4.92 (AUROC 0.76; 95% CI 0.71–0.82)CRP at week 6 (AUROC 0.75; 95% CI 0.70–0.81) and 8 (AUROC 0.76; 95% CI 0.71–0.82)	
Kassouri et al. (41)	Retrospective	N/A	CD refractory or intolerant to at least one anti-TNF therapy	29 UST and 71 vedolizumab	Effectiveness of third line biologic therapy and surgery-free survival	**Surgery-free survival (UST and vedolizumab combined)** Ileal (OR 9.0; 95% CI 1.0–81.9)Ileocolonic (OR 5.3; 95% CI 0.7–39.4)Prior adalimumab and infliximab exposure (OR 2.2; 95% CI 0.9–5.1)	**-**

*CD, Crohn's disease; CI, confidence interval; HBI, Harvey-Bradshaw index; IM, immunomodulator; OR, odds ratio; N/A, Not available; SES-CD, Simple Endoscopic Score-Crohn's Disease; TNF tumor necrosis factor*.

Important sociodemographic aspects, including gender and ethnicity are also attractive patient-related characteristics to consider. Again, subjects randomized to receive 6 mg/kg and 130 mg in the UNITI trials showed an improved clinical response rates between females and white patients compared to other races grouped together (20). Two observational cohorts support this finding, with reduced rates of combined response and remission rates after 24 weeks (35) and 48 weeks (39) in male patients, whilst the remaining short and long-term studies have not replicated this observation.

Low body weight has been associated with an improved response to anti-TNF therapy (46, 47), although controversial results have also been reported (48), and it is expected to be secondary to complex disease-related mechanisms that can influence pharmacokinetics. The currently approved loading dose of UST consists on a weight-based infusion of 260, 390, or 520 mg in patients 55, 56–85, or >85 kg, respectively. During the induction period with the initially approved dosing strategy, patients <60 kg of body weight receiving 90 mg subcutaneous UST showed a similar trend toward improved clinical response rates at week 8 (49). Consistent results had been observed in the UNITI program, were subjects in both treatment arms-−6 mg/kg and 130 mg iv—with low body weight showed improved clinical response rates in the short-term (20). Recent results from the Dutch IBD cohort have confirmed this trend, as body mass index was inversely correlated with the corticosteroid-free clinical remission at week 52 (40). Patient populations across different countries can have important differences, but body weight is a readily available information that could be easily implemented in clinical practice.

### Disease-Related Factors

Some factors associated with the characteristic of the disease should be also considered when starting UST therapy. Disease extension is one of the main items included in the Montreal classification and it defines one of the most important characteristics of the disease (50). Thus, it is of great importance to evaluate if it is associated with the response to certain immunosuppressive or biologic agents. The presence of lesions in the ileum and colon was shown to be associated with improved clinical response rates in those patients receiving 130 mg or 6 mg/kg UST in the UNITI trial, compared to placebo (OR, 2.8; CI 95%, 1.7–4.7, and OR, 5.0; CI 95%, 2.8–8.9, respectively) (20). In a Canadian retrospective cohort, Ma et al. described improved steroid-free clinical response and remission rates in ileocolonic CD (OR, 2.41; 95% CI, 1.01–5.79) (25). Further analysis from the same group were in line with their previous findings, as ileocolonic disease was associated with lower rates of loss of response during follow-up (OR, 0.26; 95% CI, 0.1–0.68) (26). Favorable results have been observed also when the disease is limited to the colon (26, 32). In contrast, the recent experience reported including 407 patients from Spain showed opposite results, as ileocolonic and colonic disease extension were associated with lower clinical response rates at week 26 (OR, 0.56 95% CI, 0.32–0.96, and OR, 0.34 95% CI, 0.16–0.69, respectively) (31).

Another important aspect that can significantly influence the response to biologics is the presence of penetrating or stricturing complications. It would be expected that patients who have shown a progression of the disease to a B2/B3 phenotype will have established and irreversible bowel damage that will be more difficult to control with medical therapy (51). In the UNITI-1 and UNITI-2 cohorts, 9–12% and 8–12% of patients had bowel strictures at baseline, while 18–20% and 15–16% had active fistulas (but there is no data available about type or location of the fistulas), respectively (20). No *post-hoc* analysis are available from these subgroups, hence data can be obtained only from observational studies. Both analysis performed in the Canadian cohort demonstrated that UST was less effective when stricturing complications have already developed, but most patients included in both cohorts received a subcutaneous induction regimen (25, 26). Similarly, a retrospective analysis of 106 CD patients receiving intravenous induction showed that penetrating complications were associated with lower rates of clinical and biochemical remission at week 48 (OR, 0.25; 95% CI, 0.07–0.89) (39). The remaining observational cohorts describing the experience across different countries with the intravenous induction did not show statistically significant differences according to disease phenotype (30–32). Additional data can be obtained from two recent analysis comparing the efficacy of UST and vedolizumab in CD (52, 53). Patients from five French university hospitals receiving either vedolizumab or UST for CD refractory or intolerant to TNF antagonists were analyzed (52). At week 48, UST was associated with higher clinical remission in patients with penetrating disease (OR, 6.58; 95% CI, 1.91–22.68). In a similar approach by the Dutch Initiative on Crohn and Colitis including 69 patients with UST and 69 with vedolizumab, there were no differences regarding the presence of intraabdominal complications at study entry (53). Therefore, accumulating evidence suggests that UST could be preferred in patients with inflammatory-predominant lesions and in those with penetrating behavior, at least after anti-TNF failure. Nevertheless, more quality data comparing the use of different biologic therapies would improve our management of patients with complicated disease.

Whereas, data about the efficacy of combination therapy with TNF antagonists has consistently shown an improvement in clinical and endoscopic outcomes (54, 55), evidence with UST or vedolizumab shows controversial results. Up to now, most of the evidence suggests no benefit of combination therapy with immunomodulators (56, 57). A recent meta-analysis including 15 studies found no improvement in clinical or endoscopic outcomes between patients receiving monotherapy or a combination of both drugs (OR, 1.1; 95% CI, 0.87–1.38; and OR, 0.58; 95% CI, 0.21–1.16, respectively) (57). Therefore, current evidence do not support a clear benefit of these strategy, but as UST is frequently used in refractory patients this decision should be carefully balanced in an individual basis.

Perianal fistulas and abscesses are severe complications that can lead to significant morbidity and reduced quality of life (58, 59). Up to 25% of patients develop perianal fistulas in the long-term, with a cumulative risk of 21% after 10 years and 26% after 20 years (60). Despite of its substantial impact on quality of life, there is a lack of randomized controlled trials about the best treatment options for this disabling complication. Immunomodulators and biologic anti-TNF agents, even alone or in combination, have been the most widely used treatments for perianal fistulas (61). However, no randomized controlled trial has evaluated the efficacy of UST in perianal fistula healing (62). Data from a *post-hoc* analysis of the CERTIFI, UNITI-1, UNITI-2 studies has reported its efficacy in active perianal fistulas—observed in 11 to 16% of patients at baseline -, although the results did not describe simple and complex fistula separately (63) (Table 2). Complete fistula healing was achieved in 24% of patients receiving 130 mg/kg and in 28% with the 6 mg/kg dosing, compared to 14% in the placebo arm. Although these results suggest a beneficial effect over placebo, a systematic review and meta-analysis did not show statistically significant differences for the induction of remission [relative risk (RR) 1.77; 95% CI 0.93–3.37] (66). However, this analysis included data only up to December 2016, so information from more recent cohorts may include additional and has the potential to obtain different conclusions. Data from uncontrolled real-world studies have reported heterogeneous results on fistula response and closure rates (23, 24, 64, 65). In 148 patients with active perianal disease included in a observational cohort from the GETAID, 39% achieved treatment success with UST (64). In this cohort, no predictive factors were associated with the main outcomes, and only the number of prior anti-TNF agents (≥3 drugs) showed a trend toward a reduced response rate. No additional predictive factors have been associated with fistula response or healing in real-world experience reported so far (23, 24, 38, 64, 65).

**Table 2:** Summary of studies evaluating the efficacy of ustekinumab for perianal complications of Crohn's disease.

**References**	**Study design**	**No perianal fistula**	**Type of fistula**	**Endpoint**	**Predictors of response**
Khorrami et al. (23)	Retrospective	18	N/A	Clinical efficacy by physician assessment	None observed
Sands et al. (63)	RCT	69 (1 mg/kg or 130 mg)70 (6 mg/kg)	N/A	Fistula response and complete fistula resolution	None observed
Wils et al. (38)	Retrospective	9	N/A	Clinical efficacy by physician assessment	None observed
Chapuis-Biron et al. (64)	Retrospective	207 (71% active)	N/A	Clinical success at 6 months	≥3 prior anti-TNF agents (OR 0.4; 95% CI 0.15–1.08; *p* = 0.056)
Attauabi et al. (65)	Retrospective	18	56% complex	Fistula response and remission at week 8, 24, and 52	None observed

*N/A, not available; RCT, randomized controlled trial*.

### Endoscopic and Histologic Factors

Increasing evidence supports the impact of mucosal and histologic healing in UC, as it has been extensively demonstrated that the resolution of the mucosal lesions improves the long-term clinical outcomes (67). Nevertheless, data supporting the influence of healing endoscopic lesions in CD is favorable, but the evidence is still more limited (68, 69). A recent systematic review with meta-analysis has shown increased clinical remission rates, but not influence on surgery risk (68). The current definition of mucosal healing suggested by the 2015 STRIDE recommendations is the resolution of ulcers at ileocolonoscopy or cross-sectional imaging (70), as it has been previously defined in the SONIC (54), ACCENT (7) and EXTEND (71) trials. The most frequently used scores are the Crohn's Disease Endoscopic Index of Severity (CDEIS) and the Simple Endoscopic Score for Crohn's Disease (SES-CD) (72, 73). However, no definite endpoints or endoscopic scores were included in the STRIDE statements based on these score (70), but the IOIBD has proposed the use of a SES-CD ≤2 (74).

No clear data can be obtained from the initial reports from the developing program of UST about potential endoscopic predictors of response (20, 49). However, a recent *post-hoc* analysis of the UNITI-1, UNITI-2, and IM-UNITI trials evaluating histologic disease activity also included some endoscopic outcomes (43). Here, Li et al. observed that baseline SES-CD inversely correlated with the histologic disease activity at week 8 (OR, 0.18; 95% CI, 0.042–0.321). The relationship between response rates and endoscopic disease severity has been also observed in real-world studies (30, 31, 33). Iborra et al. evaluated the short and long-term clinical and endoscopic response among patients included in the ENEIDA registry (30, 31, 75). Conversely, after 14 weeks of treatment endoscopic severity at baseline was negatively associated with clinical remission rates (OR, 0.08; 95% CI, 0.01–0.37) (30), and this observation was further confirmed in their follow-up at 52 weeks (OR, 0.35; 95% CI, 0.16–0.71) (31). There is only one prospective observational study that has reported real-life experience about this outcome (33). In this study, Murate et al. found that clinical response at week 24 was more frequently observed in subjects with lower SES-CD at baseline (cut-off <13) (33).

In the future, it is expected that routine assessment of endoscopic disease activity or even surrogate markers of mucosal colonic lesions will help us in the decision making process. Meanwhile, the evaluation of endoscopic severity in CD remains as an important unmet need for the stratification and follow-up assessments during medical therapy.

### Biomarkers

Even though some studies have suggested a more favorable response to UST in patients with more severe disease (25, 26), no clear conclusion can be obtained from disease scores or biomarkers associated with a specific immune pathway. No association has been observed between C-reactive protein and clinical outcomes in most of the observational cohorts (18). Potential cut-off values of C-reactive protein and additional biomarkers have been analyzed with data from UNITI-1, UNITI-2, and IM-UNITI (44). Using machine learning algorithms, some biomarkers were able to identify non-responders to UST after 42 weeks of therapy. Interestingly, lower CRP levels at week 3, 6, and 8, higher serum UST through levels to CRP ratio and increased albumin levels were associated with increased treatment success rates. A prospective observational cohort from Japan recently found that responder to UST at week 8 showed higher TNF-α concentrations at baseline (33). Moreover, serum TNF-α levels in responders were significantly decreased during anti-IL12/23 therapy. No additional serum biomarkers associated with UST response have been identified.

Results about the probability of developing loss of response to anti-TNF drugs according to specific genetic variants have shown promising results (76, 77). Those patients carrying HLA-DQA1^*^05 are at a higher risk of immunogenicity during infliximab or adalimumab therapy in patients with CD. Currently there are no published data on the possible influence of genetic factors in the response to UST, but results on this topic are awaited.

### Microbial Markers

Gut microbiota plays an essential role in the pathogenesis of IBD. Hence, it would be plausible to find a relationship with treatment response and it may even be useful as a predictive factor of response to some medical therapies. In CD, data about the influence of specific components of the fecal microbiota on treatment outcomes are still scarce (78). In a subset of patients with moderate-severe CD refractory to TNF antagonists participating in the phase 2b clinical trials of UST, Doherty et al. found that microbial signatures were associated with treatment response or remission (CDAI decrease ≥100 points or below 150 points, respectively) (79, 80). Interestingly, the predictive model performed better than clinical data alone, and the combination of both data sets did not improve significantly the area under the curve over the microbiome data by itself. Responders at week 6 had significantly different baseline α and β-diversity than subjects with active CD. *Bacteroides* and *Faecalibacterium* were the two more abundant genus in those subjects with a better treatment response. The presence of *Faecalibacterium, Blautia, Clostridium* XIVa, *Ruminococcaceae*, and *Roseburia* was also associated with in clinical remission at week 6.

### Pharmacokinetics

Anti-TNF trough and anti-drug antibody concentrations are associated with improved outcomes in IBD (81–83). Indeed, therapeutic drug monitoring has been evaluated in multiple clinical trials and observational studies in the management of patients showing a loss of response to anti-TNF agents (84–86). Despite the increasing evidence toward the utility of drugs levels with these agents, data on the optimal drug concentrations and anti-drug antibodies thresholds with novel biologics have been less extensively explored (87). In fact, there is currently scarce comprehensive data about UST pharmacokinetics and exposure-response data in CD from large, randomized, controlled trials. Table 3 summarizes the evidence of the influence of pharmacokinetics on UST response. The UNITI-1, UNITI-2, and IM-UNITI trial pharmacokinetics are the main sources exploring the relationship between trough levels and efficacy at 1 year (20, 90). A *post-hoc* analysis of the IM-UNITI cohort demonstrated an area under curve of 0.64 (*p* < 0.003) for clinical remission and UST concentrations, with an optimal cut-off of 0.8 ug/mL (90). In addition, UST concentrations >1.1 ug/mL were associated with an increased probability of C-reactive protein normalization at week 24 (52 vs. 25%, *p* < 0.0001).

**Table 3:** Studies evaluating the influence of pharmacokinetics on ustekinumab response.

**References**	**Study design**	**No of patients**	**Endpoint**	**Cut-off trough levels**	**Antidrug antibodies (%)**
**Week 2**					
Hanzel et al. (88)	Prospective observational	41	Biochemical and endoscopic remission week 24	105 μg/mL peak concentration	-
**Week 4**					
Verstockt et al. (89)	Prospective observational	86	50% decrease in fecal calprotectin week 8	>15.9	1
**Week 8**					
Adedokun et al. (90)	*Post-hoc* analysis of RCT (UNITI-1, UNITI-2 and IM-UNITI)	701	Clinical remission week 8	3.3 μg/mL	2.3
Verstockt et al. (89)	Prospective observational	86	Biological remission week 8	>7.2 μg/mL	1
Verstockt et al. (89)	Prospective observational	86	50% decrease in fecal calprotectin week 8	>4.2 μg/mL	1
Soufflet et al. (91)	Prospective observational	51	Corticosteroid-free clinical and biochemical remission week 16	2 μg/mL	-
Thomann et al. (92)	Retrospective observational	72	Clinical response week 16	2 mg/L	-
**Week 12**					
Painchart et al. (93)	Prospective observational	72	Biological response 6 months	1.10 μg/mL	0
**Week 16**					
Soufflet et al. (91)	Prospective observational	51	Corticosteroid-free clinical and biochemical remission week 16	1.4 μg/mL	-
**Week 24–26**					
Verstockt et al. (89)	Prospective observational	86	Endoscopic response week 24	1.9 μg/mL week 24	1
Battat et al. (94)	Prospective observational and cross-sectional cohort	62	Endoscopic response	4.5 μg/mL week ≥26	0
**Week 40**					
Adedokun et al. (90)	Randomized clinical trial	1,366	Clinical remission week 44	1.4 μg/mL week 40	2.3
Liefferinckx et al. (32)	Retrospective observational	152	Clinical and endoscopic response week 8, 16, and 52	None detected	-
**Negative studies**					
Rowan et al. (95)	Prospective observational	19	Clinical response	None detected	-
Murate et al. (33)	Prospective	52	Clinical response 24 weeks	No difference in clinical response	-

The relationship between UST trough concentrations has also been investigated in a real-world setting, including anti-drug antibodies and clinical outcomes (94). Battat et al. conducted a prospective study in 62 patients with refractory CD, demonstrating a relationship between serum C-reactive protein and endoscopic improvement with UST trough concentrations >4.5 μg/mL at week 26 or beyond (94). Moreover, a recent prospective open-label cohort study including 86 patients, showed that UST concentrations ≥4.2 μg/mL at week 8 were associated with a 50% decrease in fecal calprotectin (89). Additionally, week 16 UST concentrations ≥2.3 μg/mL and week 24 concentrations ≥1.9 μg/mL were associated with endoscopic response at week 24 (89).

On the other hand, evidence regarding early UST concentrations and prediction of later outcomes in CD is limited. Recently, a prospective observational study by Hanzel et al. found that 6 of 13 patients (46%) with peak concentrations above 105 μg/mL achieved endoscopic remission, compared with only 7% among those with peak concentrations below 88 mg/mL (88). These authors concluded that therapeutic drug monitoring as early as during the first 2 weeks of initiation of UST might help stratify patients according to the probability of achieving treatment outcomes at 6 months.

In contrast to anti-TNF treatment, the immunogenicity of UST seems to be very low (<5%). The incidence of antibodies against UST was 0.2% after induction and after 1 year of treatment it was only 2.3% (using a drug-tolerant assay) in the UNITI-1, UNITI-2, and IM-UNITI trials (90). This fact suggests that combotherapy with immunomodulators may not be needed with the primary aim of reducing immunogenicity. However, as discussed above some cohorts have suggested that combination therapy could improve the clinical efficacy of UST (24–26). Nonetheless, this observation has not been confirmed in more recent cohorts and one meta-analysis (56, 57).

Finally, there are multiple factors that can influence UST trough levels in an individual patient. Higher UST exposure can be expected in patients with markers of a more limited inflammatory burden and less aggressive disease like higher albumin, lower baseline C-reactive protein, lower fecal calprotectin and no previous exposure to biological therapy (88, 89). In summary, UST concentrations have been associated with improved results in refractory patients with CD, demonstrating a favorable exposure-outcome relationship. Hence, it is expected that the increasing availability of measuring UST trough levels in clinical practice may lead to a better disease control in difficult to treat patients.

### UST Intensification Strategies

Unlike with anti-TNF agents the optimal management of loss of response to UST is not fully established. Shortening the interval of administration and also re-induction with iv UST have been described in patients after an initial inadequate response or secondary loss of response with good results (96–101). However, data about the efficacy of both strategies in patients failing q8w dosing are still scarce. Dose escalation to q4w is able to decrease Harvey-Bradshaw index and C-reactive protein levels in refractory patients (98). In a study from the Groupe d'Étude Thérapeutique des Affections Inflammatoires du Tube Digestif (GETAID) clinical response was observed in 57% of patients 2 months after reducing UST dosing interval to q4 w (100). Kopylov et al. also recently reported a European multicenter retrospective real-world study assessing the effectiveness of dose optimization to q4w or q6w, intravenous re-induction or both. At week 16, 51, and 39% of patients achieved clinical response and remission, respectively (101). The possibility of re-induction with 6 mg/kg iv UST has been evaluated in other cohorts (42, 102). Re-induction has shown to induce a significant decrease in C-reactive protein levels, with endoscopic remission in 25% of patients (42). Patients already being intensified to 4-weekly dosing can also benefit from iv re-induction, with approximately half of patients (53%) achieving clinical remission and 67% response (99). Younger patients (98) with shorter disease duration (97), no prior surgery (97), perianal disease (96), higher clinical disease activity (96, 98) and corticosteroid use (96) have shown reduced response rates to these rescue strategies. There is an ongoing study (POWER) that will compare the efficacy of q8w 90 mg sc with re-induction with 6 mg/kg iv UST in patients with loss of response to maintenance sc UST (NCT03782376).

## One Step Forward: Ulcerative Colitis

### Clinical Efficacy

UST has been recently approved by the European Medicines Agency for the treatment of UC. Data from pivotal clinical trials have shown promising results about its efficacy and safety in naïve patients and also in those previously exposed to immunomodulators or anti-TNFs. Experience in UC is still scarce in clinical practice and it comes mainly from its use as compassionate drug therapy, therefore current evidence is obtained from the pivotal clinical trials (103, 104) and small observational cohorts in subjects with refractory disease (105, 106). The UNIFI study included 642 subjects receiving induction therapy with either 130 mg or 6 mg/kg of UST, 52–54% of them with concomitant steroids at baseline, 51% previously exposed to ≥1 TNF antagonist, and 17–18% after receiving both anti-TNF and vedolizumab. The comparisons between subjects randomized to UST and those assigned to the placebo arm revealed important baseline disease characteristics as predictors of clinical response (103). Remarkably, most of the characteristics associated with clinical remission at week 8 were observed across both treatment arms. The influence of disease duration has been extensively studied in CD and specially with anti-TNF treatment. Here, patients with disease duration ≤15 years showed improved rates of clinical remission, suggesting that early intervention could be important also with UST (Table 4). Additionally, some biomarkers were also found to be predictors of higher response rates, including C-reactive protein levels <10 mg/L, fecal calprotectin >250 mg/kg and fecal lactoferrin >7.24 μg/g. Clinical remission rates were also influenced by race, as Caucasian patients showed higher probability of response in both treatment arms (OR, 3.0; 95% CI, 1.63–5.56 and OR, 3.1; 95% CI, 1.68–5.70) in the 130 mg and 6 mg/kg, respectively). Similarly, non-Asian patients demonstrated better response rates. As it was previously described in CD, additional factors including weight or smoking habits seem to influence the effect of anti-interleukin therapy. Subjects in the lowest and highest weight quartiles, non-smokers or former smokers showed a similar trend toward better treatment outcomes (103). We should interpret these findings with caution, because patients recruited in this analysis may not be a representative sample of the patient profile that will be treated with UST in clinical practice, at least during our initial experience. Nevertheless, data from pivotal trials could be used as potential predictors of response at least in the short-term and they may guide further analysis in real-world studies.

**Table 4:** Summary of current evidence on predictive factors of response to ustekinumab in Crohn's disease and ulcerative colitis patients.

**Predictive factor**	**Crohn's disease**	**Ulcerative colitis**
Age	Young	
Gender	Female	
Race	White	Caucasian, non-Asian
Weight	Low body weight	Lower and higher quartiles
Smoking habits	Active smokers	Non and prior smokers
Disease duration		Shorter duration
Disease extent		
Crohn's disease behavior	Stricturing	
Disease activity	More severe	More severe
Endoscopic severity	Lower SES-CD	
Concomitant steroids		
Previous anti-TNF		
Combination therapy		
Gut microbiota	*Bacteroides Faecalibacterium*	
C-reactive protein	Low	High
Fecal calprotectin		>250 mg/kg

*Green: positive correlation; red: inverse correlation; gray: no influence or insufficient data*.

*CRP, C-Reactive protein*.

Only two observational studies have described the efficacy and safety of UST for UC in clinical practice. Ochsenkühn et al. have reported their experience in 19 patients with UC, where no predictive factors of response were identified (105). A multicentric and observational cohort from France has been recently reported in 103 patients with active disease (106). In this cohort, patients with more severe disease activity—defined as partial Mayo score >6— or prior exposure to TNF antagonists and vedolizumab were associated with a lower probability of achieving steroid-free remission at week 12–16 (OR, 0.10; 95% CI, 0.01–0.90 and OR, 0.03; 95% CI, 0.01–0.42, respectively).

### Pharmacokinetics

Data about the influence of pharmacokinetics on the pivotal clinical trials in UC show similar findings to CD (107). Serum concentrations of UST correlated well with clinical and histological efficacy features, including normalization of inflammatory markers. The authors identified that a target concentration threshold of 3.7 μg/mL at week 8 (AUC 0.65, 95% CI, 0.61–0.69) was associated with clinical response. Importantly, 5.7% of samples demonstrated anti-drug antibodies, but 44% were transient and only 28% were considered as neutralizing. Immunogenicity to UST did not seem to impact efficacy outcomes or injection site reactions. These results may help us through the treatment algorithm of UST in patients with UC, but additional data are still needed to include drug concentration of this drug in clinical practice.

## Conclusions

CD is a chronic and disabling disease that frequently leads to irreversible bowel damage. Therefore, a relevant proportion of patients receive immunosuppressants or biologics, but complete clinical or endoscopic response is achieved only in a subset. Newer biologic therapies like UST are currently used in difficult-to-treat patients, but increasing data suggest that we can identify factors associated with higher probability of response. The individualization of UST would maximize the efficacy and costs associated to this chronic and progressive condition. This is an evolving field, but data from recent years have already demonstrated many aspects that make personalized medicine with anti-interleukin biologics closer to clinical practice.

## Author Contributions

Both authors listed have made a substantial, direct and intellectual contribution to the work, and approved it for publication.

## Conflict of Interest

IR-L has received financial support for traveling and educational activities from or has served as an advisory board member for MSD, Pfizer, Abbvie, Takeda, Janssen, Tillotts Pharma, Roche, Shire Pharmaceuticals, Ferring, Dr. Falk Pharma, Otsuka Pharmaceutical, and Adacyte. AG has served as a speaker, a consultant and advisory member for or has received research funding from MSD, Abbvie, Pfizer, Kern Pharma, Takeda, Janssen, Ferring, Faes Farma, Shire Pharmaceuticals, Tillotts Pharma, Chiesi, and Otsuka Pharmaceutical.
